# Marketization of data elements drives the cultivation of competitive advantages in export products—Based on an export technology complexity perspective

**DOI:** 10.1371/journal.pone.0342262

**Published:** 2026-02-09

**Authors:** Kongtuan Lin, Xinjie Wang

**Affiliations:** School of Economics, Fujian Normal University, Fuzhou, Fujian, People’s Republic of China; USTC: University of Science and Technology of China, CHINA

## Abstract

The policy of marketizing data factors, recognized as a pivotal institutional innovation in the digital intelligence era, serves as a crucial catalyst for the high-quality development of enterprises. The intricate nature of export technology, which reflects the high-quality development of these enterprises, interacts synergistically with data marketization, thereby establishing a virtuous cycle. Utilizing data from China’s A-share listed companies spanning the years 2012–2022, this study employs a quasi-natural experiment framework, predicated on the establishment of a data trading center, to develop a difference-in-differences model aimed at assessing the impact of data element marketization on the technological complexity of enterprise exports. The findings indicate that the marketization of data factors significantly enhances the technological complexity of firms’ exports, a conclusion that remains robust across various tests. Mechanistic analyses reveal that marketization of data factors contributes to a reduction in transaction costs, bolsters innovation incentives, mitigates innovation risks, and improves total factor productivity, thereby facilitating an increase in export technological complexity. Furthermore, heterogeneity tests demonstrate notable disparities in the effects of data factor marketization on the enhancement of export technological complexity; specifically, enterprises exhibiting high agile response capabilities, advanced levels of smart technology, deeper applications of smart technology, and those operating within high-monopoly industries experience more pronounced benefits. This research offers new empirical insights into the role of data factors in promoting the high-quality development of trade and serves as a valuable reference for governmental policy formulation.

## Introduction

Within the framework of the digital transformation of the global economy, data is increasingly recognized as the “fifth factor of production,” alongside land, labor, capital, and technology. This evolution is significantly altering the dynamics of international industrial competition and influencing the ways in which enterprises engage in the global value chain. In April 2020, the Central Committee of the Communist Party of China, along with the State Council, released the “Opinions on Constructing a More Perfect Institutional Mechanism for Market-based Allocation of Factors.” This document outlines the requirements and objectives for “accelerating the cultivation of the data factor market.” The fundamental aim of marketizing data factors is to facilitate the synergistic optimization, reuse, and efficiency enhancement of data resources, as well as to foster integration and innovation through the support of digital infrastructure. This process seeks to transform data factors into valuable resources, assets, and capital, thereby enhancing their value. Ultimately, it aims to establish an environment conducive to data circulation characterized by openness, sharing, and trading of data. The significance of marketization of data factors is evident not only in its capacity to lower information costs and transaction frictions for enterprises engaged in the international division of labor, but also through the data-driven innovation mechanism that facilitates the transition of enterprises from a “factor-input” export model to a “technology-innovation” export model.

However, existing research has largely overlooked this critical pathway in the analytical framework of export sophistication. Specifically, there is a lack of empirical verification regarding its actual effect on export upgrading, as well as a systematic explanation of its underlying mechanisms—particularly in connection with the distinctive features of data factors such as intangibility and mobility. To address these gaps, this study focuses on the following questions: First, it empirically examines the net effect of data factor marketization on export sophistication and explores potential heterogeneities. Second, it identifies key mechanistic pathways, including transaction cost reduction, innovation incentives, and improvements in total factor productivity. Finally, it employs a more rigorous identification strategy to mitigate endogeneity concerns, thereby providing a reliable theoretical interpretation and empirical evidence for how data factors drive export technological upgrading.

## Literature review

To fully understand the impact of data factor marketization on export sophistication, it is essential to first grasp the unique attributes of data as a factor of production, which distinguish it from traditional factors of production. In contrast to traditional production factors such as land and labor, data factors exhibit notable virtual characteristics [1]. The process of realizing the value of data elements is inherently complex, necessitating a series of technical processes including data collection, cleaning, analysis, and modeling. The establishment of a market for data elements is crucial for unlocking their value, as it can enhance total factor productivity and digital transformation capabilities of enterprises by improving the efficiency of data resource allocation, reducing transaction costs, and fostering innovation [2,3]. It also offers a potential micro-level explanation for the increase in export technology complexity. However, the current state of China’s data elements market remains in the nascent stages of exploration, characterized by ambiguous property rights definitions and an incomplete market standards system [4]. Presently, issues such as “data silos” and “data monopolies” significantly hinder the efficient flow of data elements, obstructing the full realization of data dividends and diminishing the positive impact of data elements on economic development [5]. In light of these challenges, enhancing the data supply and demand mechanism, refining the legal framework governing data property rights, and establishing a comprehensive market regulatory mechanism represent viable strategies to mitigate existing developmental bottlenecks and advance the marketization of data factors in China [6,7]. It also helps create a favorable institutional environment for data factor-driven upgrading of export technology.

In the context of the transformation and enhancement of the export model, the technological complexity of exports serves as a fundamental metric for assessing regional export competitiveness. This complexity not only indicates the technological sophistication and production efficiency of exported goods [8] but also signifies the extent to which the regional export structure has been optimized. The presence of high-technology export products represents a shift in the export model from a focus on “factor-input-oriented” strategies to an emphasis on “technology-innovation-oriented” approaches. This transition is shaped by a variety of multidimensional factors. Research has investigated the determinants of the rising technological complexity of exports at the national, industry, and firm levels. At the national level, traditional factors such as enhanced infrastructure development, greater trade openness, and the effective utilization of foreign direct investment (FDI) significantly contribute to the technological complexity of exports [9,10]. Concurrently, in light of the recent wave of scientific and technological advancements and industrial transformations, the enhancement of new digital infrastructure has emerged as a novel catalyst for the advancement of international trade [11]. Additionally, government policies promoting openness, such as the Belt and Road Initiative and the establishment of Pilot Free Trade Zones, alongside improvements in financial development and institutional quality, have positively influenced the technological complexity of exports. At the industry level, the transition of the manufacturing sector towards a service-oriented model, the specialization and diversification within digital clusters, and the upgrading of industrial structures have collectively driven increases in both the technological complexity of exports and the quality of export products [12–14]. At the firm level, the expansion of human capital has been shown to elevate the technological complexity of exports for processing trade firms [15]. Furthermore, manufacturing firms can enhance their value chains through technological innovation, optimization of product structures, and improvements in production processes, thereby increasing the technological complexity of their exports [16].

Existing research has explored the export of technological complexity from multiple dimensions; yet, two key controversies and limitations persist. First, divergent views exist regarding the driving logic—a debate between “factors” and “institutions.” Some studies emphasize the core role of traditional factors (such as infrastructure and human capital). In contrast, others focus on the influence of institutional environments (such as free trade zone policies and financial development). Neither side has reached a consensus on how new factors or institutions impact export technological complexity. Second, a structural imbalance exists between macro-level and micro-level analysis—most studies validate aggregate effects at the national or industrial level but fail to dissect the micro-mechanisms through which firms enhance technological complexity via specific pathways, such as data utilization and process upgrading. Crucially, they neglect to incorporate the “marketization of data as a factor of production”—a novel institutional arrangement of the digital economy era—into their analytical frameworks, creating a significant conceptual gap. This limitation creates space for theoretical expansion and academic supplementation, which this paper addresses by focusing on the interaction mechanism between data factor marketization and export technological complexity.

The marketization of data factors influences the technological composition of exports by improving resource allocation efficiency, minimizing transaction costs, and fostering technological innovation. The effective circulation of data elements enhances enterprises’ ability to respond to fluctuations in international market demand. Furthermore, the synergies derived from data-driven production have transformed the conventional manufacturing paradigm, thereby facilitating the advancement of export technologies [3]. This study focuses on A-share listed companies in Shanghai and Shenzhen from 2012 to 2022, utilizing a range of data including international trade statistics, global per capita GDP figures, information on listed enterprises, and various industry-specific data. By examining the marketization of data elements as a focal point, this research treats the establishment of a data element trading center as a quasi-natural experiment. It investigates the channels and specific effects through which the marketization of data elements influences both the export capabilities and technological complexity of enterprises. The findings indicate that the marketization of data factors significantly enhances the technological complexity of export activities among enterprises, a conclusion that remains robust across multiple tests. Mechanistic analysis reveals that the marketization of data factors contributes to increased export technology complexity by leveraging the value of data through mechanisms such as reduced transaction costs, enhanced innovation incentives, risk mitigation in innovation, and improvements in total factor productivity. Furthermore, heterogeneity analysis demonstrates notable variations in the impact of data factor marketization on export technology complexity, with enterprises exhibiting high responsiveness, advanced levels of smart technology, deeper integration of smart technology, and those operating in highly monopolistic industries experiencing more pronounced benefits. The potential marginal contribution of this paper manifests primarily in two dimensions: theoretical and empirical contributions.

(1) Theoretical contribution: From a research perspective, beyond mainstream areas such as traditional production factors and digital transformation, further attention should be directed toward leveraging the value of data elements to enhance the international competitiveness of export products and drive high-quality transformation and upgrading of export models. This paper conducts exploratory research on technological trade upgrading from the perspective of efficient circulation in data factor markets, examining the causal pathways through which market-oriented development of data factors influences export technological complexity. In terms of research content, studies on data factor marketization and export technological complexity remain largely isolated, lacking a systematic examination of the synergistic mechanisms between unlocking data value and cultivating high-value-added products. This paper introduces the emerging institutional arrangement of data factor marketization, integrating it with export technological complexity within a unified analytical framework. It systematically proposes, for the first time, the mechanisms of reduced transaction costs, innovation incentives, and risk smoothing, as well as enhanced total factor productivity, thereby enriching theoretical research on data factors in the field of trade technological upgrading.
(2) Empirical Contributions: Methodologically, empirical studies grounded in robust theoretical analysis remain scarce and fragmented. Measuring the impact of data factor marketization reforms on firms’ export technological complexity to uncover the mechanisms through which data factors drive export technological upgrading holds significant practical relevance. This paper innovatively treats the establishment of data trading centers as a quasi-natural experiment. By constructing a progressive difference-in-differences model, it identifies the promotional effects of data marketization on firms’ export technological complexity, providing practical evidence for deepening data marketization reforms and advancing the transformation and upgrading of export patterns.

The remainder of this paper is arranged as follows: Part II is the characteristic facts and research hypotheses, which introduces the characteristic facts of data factor marketization in China and explains the mechanism of data factor marketization on export technological complexity, and finally puts forward the corresponding hypotheses based on the analysis of the mechanism; Part III is the data description and research design, which describes the data sources and processing, empirical model construction, variable measurement and descriptive statistics; Parts IV, V and VI are empirical analysis, based on the benchmark regression model, which analyzes the impact, mechanism and heterogeneity of data factor marketization on export technical complexity; Part VII is the research conclusion and policy recommendations.

## Characterization facts and research hypotheses

### Characteristic facts of data factor marketization in China

The evolution of China’s data factor market can be categorized into two distinct phases. The initial phase, spanning from 2009 to 2013, represents the nascent development of the data factor market. During this period, while a fundamental technical infrastructure was established, the absence of a cohesive framework governing rights, transactions, and regulatory measures led to suboptimal allocation of data resources and chaotic market practices. This phase is marked by a juxtaposition of technology-driven local initiatives and ineffective growth, occurring in the absence of a structured system.

The second phase pertains to the evolution of the data factor market, spanning from 2014 to the present. In 2014, the term “big data” was incorporated into the government work report for the first time. Subsequently, in 2015, the “Outline of Action for Promoting the Development of Big Data” underscored the recognition of data as a fundamental strategic resource for the nation. In 2020, the Central Committee of the Communist Party of China (CPC) and the State Council released the “Opinions on Constructing a More Perfect Institutional Mechanism for Market-based Allocation of Factors.” In April of the same year, they further articulated the need to “accelerate the cultivation of the data factor market” through the issuance of additional opinions on enhancing the institutional framework for market-based factor configuration. Most recently, in July 2024, the Decision of the Central Committee of the Communist Party of China on Further Comprehensively Deepening Reform and Promoting Chinese Modernization, articulated during the Third Plenary Session of the 20th CPC Central Committee, explicitly advocated for the development of a national integrated technology and data market.

As of 2022, a total of 49 data trading centers have been established throughout the nation. These existing data trading platforms can be categorized into three primary types based on their construction leadership. The first category includes platforms led by local governments, exemplified by entities such as the Guiyang Big Data Exchange and the Wuhan East Lake Big Data Exchange Center. The second category encompasses enterprise-led trading platforms, which provide data products or services, typically leveraging substantial internal data resources or proprietary technologies as their competitive advantage. The third category consists of trading platforms that function as industrial alliances, such as the Zhongguancun Big Data Industry Alliance and the Heilongjiang Province Big Data Industry Association.

In terms of regional development patterns, China’s data trading centers exhibit pronounced spatial clustering, with the Beijing-Tianjin-Hebei region, the Yangtze River Delta, and the Pearl River Delta capitalizing on market-oriented advantages and institutional innovations to prioritize the development of data trading and value-added services. Conversely, the central and western regions leverage their inherent energy and computational resources to foster a differentiated and synergistic industrial ecology.

The establishment of data trading centers has created an institutionalized platform for the marketization of factors, effectively lowering the barriers and costs for enterprises to access data, thereby enhancing their operational performance and productivity. Furthermore, by utilizing the data ecological network established by these trading centers, enterprises can achieve more efficient collaborative innovation and improve the accuracy of their decision-making processes. It is important to note that data trading platforms serve not merely as transactional intermediaries; rather, they facilitate the open sharing and co-creation of data value among various stakeholders through standardized circulation protocols and synergistic mechanisms, both on-site and off-site.

## Theoretical analysis and research hypothesis

### Technical complexity and data elements for marketization

In the context of the swift advancement of the digital economy, the marketization of data elements has emerged as a significant catalyst for increasing the complexity of export technologies. This phenomenon is achieved through the reconfiguration of the innovation ecosystem and the optimization of industrial structures. The influence of market competition compels firms to consistently enhance their production and innovation processes, thereby reinforcing the impetus for upgrading the export structure. This dynamic ultimately facilitates a synergistic advancement in both regional innovation efficiency and the complexity of export technologies. At the level of industrial structure, the marketization of data elements has not only facilitated the emergence of new industries, exemplified by advanced information technology and high-end equipment manufacturing, but has also catalyzed the transformation and enhancement of traditional industries through digital transformation. This process has resulted in traditional export products being increasingly integrated with advanced digital technologies and innovative components. Significantly, within the context of factor allocation, the data factor market establishes an effective price signaling mechanism that directs the concentration of capital, talent, and other essential production factors towards high-tech industries. This process not only bolsters the research and development capabilities associated with high-tech products but also improves the overall efficiency of resource allocation. Furthermore, it facilitates the transition of exported goods from being labor-intensive to knowledge-intensive, ultimately leading to a structural enhancement in the technological complexity of exports. In light of this, we propose the following research hypothesis:

**H1:** The marketization of data elements is effective in increasing the technological complexity of firms’ exports

### Transaction cost reduction mechanisms

A critical objective in the development of a market-oriented framework for data elements is to diminish the barriers for market participants in accessing data, thereby enhancing the equity and rationality of data availability. This standardized approach not only assists organizations in mitigating informational disadvantages but also substantially lessens the decision-making risks linked to information asymmetry. The benefits of this enhancement are evident in both the improved efficiency of information dissemination within the organization and the reduced level of information asymmetry between the firm and the external market. Consequently, enterprises are better positioned to accurately discern market trends and consumer preferences from extensive datasets, both standardized and non-standardized, thereby elevating the quality of their decision-making processes [17]. The marketization of data factors facilitates enterprises in accurately understanding the technical standards and quality requirements associated with the high-end segments of the global value chain, thereby enabling targeted technological innovation. In this context, enterprises can refine their research and development (R&D) strategies and strategically allocate their limited resources towards high-tech, complex products that possess international competitiveness. This approach systematically enhances the technological sophistication and market competitiveness of their export offerings. From an organizational change perspective, the marketization of data elements not only compels enterprises to undergo digital transformation but also necessitates a restructuring of their coordination mechanisms. To effectively adapt to the evolving data landscape and optimize the value derived from information technology, enterprises must refine their organizational structures and management models [18]. The marketization of data within factor markets facilitates more efficient resource allocation for technological research and development by reducing information asymmetries and necessitating organizational transformations within enterprises [19]. The establishment of a data-driven factor market not only reduces the research and development timeline for essential technologies but also fortifies the institutional framework that supports ongoing innovation within enterprises by enhancing organizational and managerial efficiency. This, in turn, contributes to the advancement of the technological sophistication of export products. Consequently, we put forth the following research hypothesis:

**H2:** The development of data elements for marketization can indirectly enhance the technological complexity of exports by reducing information asymmetry and management costs.

### Innovation incentives and risk smoothing mechanisms

Based on the signaling theory [20], The creation of the Data Exchange signals the government’s dedication to fostering the high-quality advancement of the digital economy, representing an institutional innovation that increases the technical sophistication of exports. In particular, the special subsidy initiatives—such as research and development (R&D) subsidies and tax incentives—introduced by local governments to facilitate the establishment of data exchange centers have established a robust system of innovation incentives. This system not only substantially mitigates the costs and risks associated with enterprises engaging in R&D for technologically complex products but also enhances their innovation outcomes by improving the efficiency of innovation resource allocation. In addition, the marketization of data elements significantly enhances enterprises’ capacity to manage innovation-related risks. In the realm of information processing, the Data Elements Market improves decision-making capabilities by supplying timely and precise technical information pertinent to the industry. This enables firms to swiftly comprehend technological development trends and market fluctuations, thereby effectively mitigating potential innovation risks. The implementation of unified data standards has standardized information exchange among enterprises, thereby enhancing the allocation efficiency of innovation resources and minimizing research and development errors stemming from information distortion. This framework contributes to the technological complexity of firms’ exports by diminishing innovation risks. Furthermore, the rapid expansion of the data factor market has facilitated the emergence of new cross-industry innovation consortia, which have successfully integrated various innovation elements throughout the industrial chain by establishing standardized data matching and resource-sharing mechanisms. This networked innovation structure enhances the technological complexity of exports by bolstering the stability and risk tolerance of firms [21]. In light of this, we put forth the following research hypothesis:

**H3:** The development of a market-oriented data element system can leverage R&D subsidies and policy expectations to stimulate corporate investment in innovation and technology commercialization. This approach simultaneously enhances enterprises’ risk resilience and R&D stability, thereby indirectly elevating the technological complexity of exported products.

### Total factor productivity enhancement mechanisms

The marketization of data factors enhances the technological sophistication of firms’ exports by increasing total factor productivity within the manufacturing sector. Total factor productivity, which measures output relative to input, is conceptualized within the Solow residual accounting framework as the “efficiency dividend” of total output attributable to technological advancements, institutional modifications, and other influences beyond mere factor inputs. This metric serves as a clear indicator of the efficiency with which resources are employed [22]. The ongoing enhancement of data elements related to the optimization of production processes has markedly improved the accuracy of management decisions and the efficacy of technological innovation. Consequently, the advancement of big data has played a significant role in augmenting the total factor productivity of manufacturing enterprises [23]. When this enhancement effect is applied to the domain of international trade, it is observed that total factor productivity encourages firms to direct their exports towards products characterized by high technological content and significant value addition. This shift consequently fosters an increase in the technical complexity of the exports produced by these enterprises. In light of this, we propose the following research hypothesis:

**H4:** The development of data elements for marketization can indirectly enhance the technological complexity of exports by optimizing production processes and improving decision-making efficiency, consequently increasing total factor productivity and promoting the upgrading of export products.

**Fig 1:**
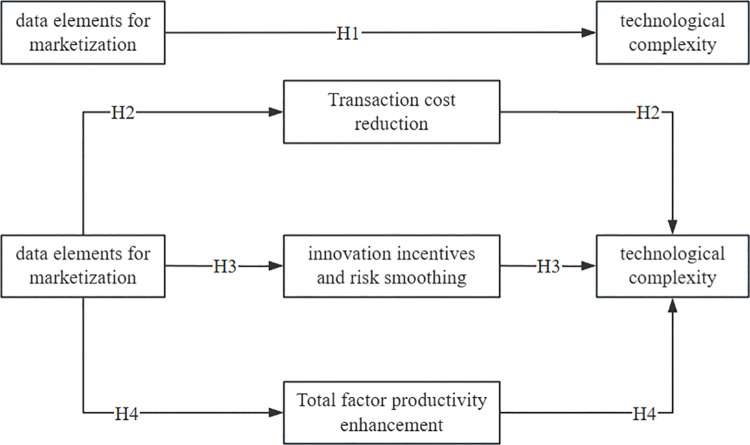
Schematic Diagram of the Theoretical Mechanism.

As shown in Fig 1, the marketization of data elements collectively enhances the technological complexity of corporate exports through three pathways: reducing transaction costs, incentivizing innovation and smoothing risks, and boosting total factor productivity.

## Data description and research design

### Data sources and sample selection

This study investigates the impact of the construction of digital factor marketization on the complexity of export technology. The research focuses on A-share listed companies in Shanghai and Shenzhen from 2012 to 2022. The data utilized in this analysis is categorized into four primary components: inter-country trade data, global per capita GDP data, data pertaining to listed companies, and industry-specific data. The inter-country trade data is sourced from the CEPII database, while the global per capita GDP data is obtained from the World Bank’s World Development Indicators (WDI) database. Data on listed enterprises is derived from the Wind database, and industry data is extracted from the China Industrial Statistical Yearbook. In accordance with established practices in prior literature, the sample data undergoes several processing steps: the exclusion of listed companies under special treatment, the removal of samples from the financial sector, and the elimination of samples with missing key variables. Consequently, this research yields a total of 20,846 annual observations of listed companies for analysis.

### Model Setting and Variable Definition

The establishment of the Data Factor Exchange represents a significant initiative aimed at advancing the marketization of data factors [2], The subject exhibits notable exogenous characteristics. The decisions regarding site selection are predominantly influenced by macroeconomic factors, including regional development strategies and infrastructure, rather than by the enterprises’ operational conditions or innovation outcomes. This policy characteristic renders it challenging for firms to predict whether their locations will be chosen, and it complicates their ability to influence site selection decisions. Consequently, this situation creates a quasi-experimental framework that facilitates the examination of the effects of data factor trading centers on the technical complexity of enterprises’ exports, thereby effectively addressing the issue of endogeneity. In this study, a Staggered Differences-in-Differences (Staggered DID) approach is employed, with the regression equation formulated as follows:


$$\text{ln}\;{ESI}_{it+\text{1}}=\alpha_{\text{0}}+\alpha_{\text{1}}{{\text{T}reat}_{i}\times \text{P}ost}_{t}+\alpha_{\text{2}}X_{it}+\delta_{i}+{\;\theta }_{t}+\varepsilon_{it}$$ (1)

where, considering the lagged effect of the establishment of digital factor exchange centers, ${ESI}_{it+\text{1}}$ is used to represent the natural logarithm of the level of export technological complexity of enterprise i in year t+1. ${\text{T}reat}_{i}$ is a grouping variable used to determine whether the region where enterprise i is located has set up a data factor exchange center, and takes the value of 1 if it is, otherwise it is 0. ${\text{P}ost}_{t}$ is a time grouping variable. This paper assigns a value of 1 to the year in which enterprise i’s region establishes a data factor trading center and subsequent years, and 0 otherwise. $X_{it}$s a series of control variables at the enterprise level, $\delta_{i}$ is an enterprise fixed effect, and $\begin{array}{c} {\;\theta }_{t} \end{array}$ is a time fixed effect to control for the impact of time−varying factors on the complexity of an enterprise′s export technology. $\varepsilon_{it}$ is a random error term.

### Export technology complexity

Export technological sophistication serves as a crucial indicator for measuring product quality. The development of this metric originated with Michaely’s Technological Sophistication Index (TSI), which posits that a country’s export technological content positively correlates with its per capita income level [24]. To overcome potential estimation biases in small-country samples, Hausmann [8] further developed the PRODY index. By incorporating a product’s share of a country’s total exports as an internal weighting factor, this approach enhances the robustness of the indicator. Considering the unique characteristics of China’s export trade, some scholars have modified this model. For instance, Xu noted that the index fails to identify quality differences within the same product category effectively. Although Xu attempted to construct the QEPXY indicator based on unit value to capture the quality dimension, this approach faces theoretical challenges in the Chinese context—price differences in Chinese exports often reflect cost advantages rather than genuine quality gradients, undermining the logic of using price as a proxy for quality.[25]

Existing research on measuring the technological complexity of Chinese exports still exhibits a “quality dimension” measurement blind spot. Price signals are susceptible to cost-related distortions, while the PRODY index itself struggles to reflect genuine technological efficiency in production processes. Therefore, this paper adopts the export technological complexity measurement framework proposed by Hausmann, incorporating improvements from Dai and Fang [23]. It introduces a “firm-level total factor productivity (TFP)” adjustment to the PRODY index to measure export technological complexity at the manufacturing industry level. TFP directly measures the comprehensive technological efficiency and progress level within the production process, circumventing distorted price signals. By focusing on the “production efficiency” dimension, it provides a more robust theoretical foundation for understanding and measuring the technological complexity of China’s exports.

Export technological complexity is first measured for specific product q:


$${\text{PRODY}}_{q}=\sum_{c}\left(\frac{x_{cq}/X_{c}}{\sum_{c}\left(x_{cq}/X_{c}\right)}\right)Y_{c}$$ (2)

In Eq. (2), q denotes a product in HS92 quintile 6, c represents a country or region, x denotes the value of product exports, X is the total amount of exports, and Y denotes the level of GDP per capita.

On the basis of the technical complexity of product exports, the technical complexity of industry exports is calculated as.


$$\;{\text{EXPY}}_{i}=\sum_{q}\left(\frac{x_{iq}}{X_{i}}\right)\times {\text{PRODY}}_{q}$$ (3)

In Eq. (3), xiq /Xi denotes the share of exports of product q in industry i in total exports of industry i.

Next, the LP method is used to estimate the firm’s total factor productivity by setting the firm’s production function as [26,27]:


$$Y_{i}=A_{i}L_{i}^{\alpha_{\text{1}}}K_{i}^{\alpha_{\text{2}}}M_{i}^{\alpha_{\text{3}}}$$ (4)

Based on the above function, the estimated model of total factor productivity is obtained:


$$\text{ln}\;Y_{i}=\alpha_{\text{0}}+\alpha_{\text{1}}\;\text{ln}\;L_{i}+\alpha_{\text{2}}\;\text{ln}\;K_{i}+\alpha_{\text{3}}\;\text{ln}\;M_{i}+\xi_{i}$$ (5)

$$TFP_{i}=\text{ln}\;Y_{i}-\alpha_{\text{1}}\;\text{ln}\;L_{i}-\alpha_{\text{2}}\;\text{ln}\;K_{i}-\alpha_{\text{3}}\;\text{ln}\;M_{i}$$ (6)

In the above formula, the output variables: Y  represents the output of the enterprise, measured by operating income;L is the labor input, which covers the compensation paid by the enterprise to regular employees, non−regular employees, managers and owners of the enterprise during the year; K  represents the capital input of the enterprise, measured by the input of fixed assets; M  refers to the intermediate inputs, measured by the input of current assets; A  represents the level of technology, and TFP  is the total factor productivity of the enterprise.

The total factor productivity of the enterprise is used to adjust the industry’s export technology complexity indicator to obtain the export technology complexity at the enterprise level, Eq:


$$\;{\text{ESI}}_{i}=\frac{{\text{TFP}}_{\text{i}}}{{\text{TFP}}_{\text{j}}}\times {\text{ESI}}_{j}$$ (7)

Where ${\text{ESI}}_{i}$ denotes the export technology complexity of firm i, ${\text{TFP}}_{i}$ denotes the total factor productivity of firm i, ${TFP}_{j}$ denotes the average total factor productivity of firm  j in firm i’s industry.

### Control variable

This paper examines the influence of financial position and governance capacity on the complexity of export technology within enterprises, drawing upon existing research. The analysis incorporates several key variables: return on equity (ROE), defined as net profit relative to average net assets; total asset turnover (ATO), which is operating income as a ratio of average total assets; (Cashflow), represented as the ratio of net cash flow from operating activities to total assets; inventory ratio (INV), calculated as the ratio of net inventory to total assets; and board size (Board), quantified as the natural logarithm of the number of members on the board of directors. Descriptive statistics for the primary research variables are presented in Table 1.

**Table 1:** Descriptive statistics.

Variable	Observations	Mean	Std.dev.	Min.	Max.
LnESI	20846	10.7813	0.1598	10.3179	11.0449
DID	20846	0.2836	0.4507	0.0000	1.0000
ROE	20846	0.0664	0.2332	−14.8186	2.3789
ATO	20846	0.6690	0.4039	0.0056	7.7880
Cashflow	20846	0.0529	0.0697	−0.6581	0.8385
INV	20846	0.1376	0.0880	0.0000	0.8606
Board	20846	2.1047	0.1913	1.3863	2.8904

## Empirical results and analysis

### Benchmark regression results

The benchmark results are presented in Table 2. Column (1) displays the regression outcome without the inclusion of control variables, while controlling for both time and firm fixed effects. At this stage, the coefficient for the data factor marketization construction is significantly positive. In column (2), the regression results reflect the inclusion of firm-level control variables while only controlling for time fixed effects, yielding a regression coefficient of 0.0047, which remains significantly positive at the 10% significance level. Column (3) presents the results of controlling solely for firm fixed effects after incorporating firm-level control variables, where the coefficient for data factor marketization construction increases and retains significance at the 1% level. In column (4), following the addition of firm-level control variables, the coefficient for data factor marketization construction is reported as 0.0046, which is also significantly positive at the 1% level. Although the absolute value of the difference-in-differences (DID) coefficient is small (0.0046), its economic significance is clear and substantial. Since the dependent variable is the logarithmic form of export technological complexity (LnESI), following the interpretation rules for log-log models, this DID coefficient can be approximated as an elasticity effect: the implementation of data factor marketization policies drives an average increase of approximately 0.46% in export technological complexity. From an economic perspective, this impact holds significant value. Particularly at the macro industrial policy level, this effect generates cumulative amplification within the sample group of enterprises. This indicates that while the role of data factor marketization in export structure upgrading constitutes a “marginal improvement, “ its direction remains consistently positive and statistically significant. This provides robust empirical support for the positive effects of the policy

**Table 2:** Benchmark regression results.

Variables	(1) LnESI	(2) LnESI	(3) LnESI	(4) LnESI
DID	0.0036** (0.0018)	0.0047* (0.0024)	0.0748*** (0.0019)	0.0046*** (0.0018)
Controls	No	Yes	Yes	Yes
Time FE	Yes	Yes	No	Yes
Firm FE	Yes	No	Yes	Yes
Observations	20846	20846	20846	20846
R-squared	0.8983	0.1058	0.8532	0.9017

Note: ***, **, and * indicate significant at the 1%, 5%, and 10% levels, respectively; values in parentheses cluster standard errors. Same as below

Collectively, these findings suggest that the regression coefficient for data factor marketization construction is consistently significantly positive, irrespective of the inclusion of control variables or the simultaneous control of time and firm fixed effects, thereby indicating its potential to enhance the technological complexity of firms’ exports.

### Robustness check

#### Parallel trend test.

Prior to employing the double difference method, it is essential to verify that both the treatment and control groups fulfill the requirements of the parallel trend assumption. This study aims to assess whether the treatment and control groups adhere to this assumption prior to the establishment of the digital trading platform and its subsequent effects.

**Fig 2:**
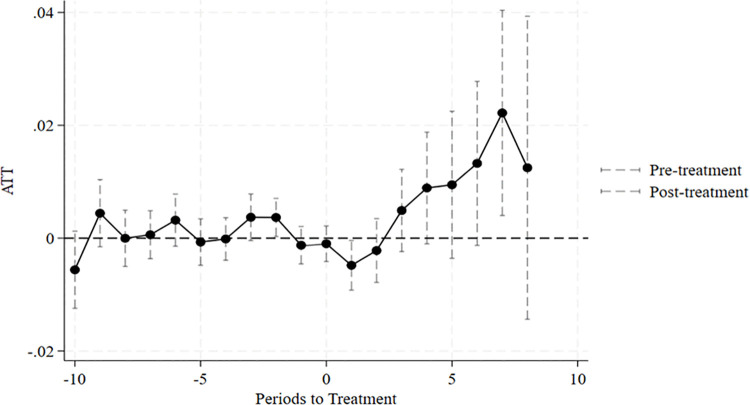
Parallel trend test.

As shown in Fig 2, the difference-in-differences estimation indicates that the ATT is not significant before the policy implementation, satisfying the parallel trend assumption. In the initial phase of policy implementation (years 0–2), the effect does not emerge, which may be attributed to the platform construction period and policy time lag; in the later stage, the ATT becomes significantly positive, confirming that the marketization of digital factors has a gradual promoting effect on enhancing the technological complexity of enterprise exports.

### Propensity score matching-double difference test

The creation of the data trading platform may be susceptible to sample selection bias due to its non-random nature. To mitigate the potential influence of other disparities between the firms in the treatment and control groups on the technical complexity of exports, we employ Propensity Score Matching with Difference-in-Differences (PSM-DID) estimation as a robustness check. In the matching procedure, we utilize a 1:1 nearest-neighbor matching approach to refine the samples from both the experimental and control groups, thereby minimizing pre-existing differences in their characteristics prior to the establishment of the data factor trading center. The regression results presented in Column (1) of Table 3, following PSM matching, indicate that the marketization of data factors continues to significantly enhance the technological complexity of firms’ exports, corroborating the findings from the baseline regression analysis. This result carries two important implications. First, it confirms that the policy effect observed in the baseline regression does not stem from sample selection bias caused by pre-existing differences between the treatment and control groups. Second, despite the reduced sample size after matching (N = 11,506), the model maintains high explanatory power (R² = 0.9140), indicating that the policy effect remains discernible after controlling for other confounding factors, thereby enhancing the reliability of our core findings.

**Table 3:** Robustness test results.

Variables	(1) PSM-DID	(2) Excluding municipalities	(3) Phase I	(4) Phase II
DID	0.0056* (1.6510)	0.0056*** (0.0019)		0.0360* (0.0204)
IV			0.0036*** (0.0002)	
Control	Yes	Yes	Yes	Yes
Time/firm Fe	Yes	Yes	Yes	Yes
Observations	11506	17869	17193	17193
R-squared	0.9140	0.9037		
K-P rk LM (p)				278.940 (0.00)
K-P Wald rk F				394.470 (16.380)

### Excluding municipalities

Considering that the municipalities sample exhibits more favorable external conditions regarding economic development, business environment, and policy support, this study undertakes additional regression analyses by excluding all municipality samples to mitigate the potential influence of these atypical samples on the benchmark regression outcomes. As demonstrated in column (2) of Table 3, the exclusion of municipalities reveals that the variable representing the marketization of digital factors remains significantly positive at the 1% level, thereby affirming the validity of the previous conclusion. This finding reveals that the positive effect of data factor marketization on export technological complexity is not driven by a few uniquely advantaged municipalities. On the contrary, the policy effect proves to be more robust and pronounced within the broader sample of ordinary cities.

### Instrumental variable method

To address the endogeneity problem, this paper uses the number of fixed-line telephones per 1,000 people in each city in 1984 as an instrumental variable for the data trading platform [17,28]. Firstly, as a traditional communication infrastructure, the density of fixed-line telephones is closely linked to the level of regional informatization. Historical data indicate that regions with a higher penetration of fixed telephones tend to have a stronger foundation for information exchange. Therefore, it can be inferred that the number of fixed telephones is related to the establishment of data trading platforms, fulfilling the correlation requirement for instrumental variables. Secondly, since the number of fixed telephones in 1984 is a historical fixed value, it does not directly influence the technological complexity of firms’ exports, thus satisfying the exogeneity requirement for instrumental variables. However, since the 1984 data on fixed telephones is cross-sectional, while this paper uses panel data, it adapts the interaction term between the number of landline telephones in 1984 and the number of Internet users from the previous year as a panel instrumental variable (IV) for the data trading platform. Regressions are conducted using two-stage least squares. The results presented in column (3) of Table 3 indicate that the regression coefficients of the instrumental variable on the construction of the digital factor market are significantly positive. This suggests that a higher number of landline telephones in a city correlates with an increased likelihood of establishing a data trading platform there. More importantly, the Kleibergen-Paap rk LM test (statistic = 278.94, p = 0.00) rejects the null hypothesis of underidentification, while the Kleibergen-Paap Wald rk F statistic reaches 394.47, substantially exceeding the critical value of 16.38 at the 10% significance level. These results provide strong evidence that the instruments are highly correlated with the endogenous variables, effectively mitigating concerns regarding weak instruments. Furthermore, column (4) of Table 3 shows that the regression coefficient for the second stage of the digital factor marketization construction remains positive and significant at the 10% level. This indicates that the findings of this paper are still robust after the endogeneity test. Moreover, the estimated coefficient shows a notable increase compared to the baseline results, which is consistent with the theoretical expectations of instrumental variable estimation under the Local Average Treatment Effect (LATE) framework. This evidence suggests that the policy’s impact is particularly accentuated in cities where historical communication infrastructure created favorable conditions for establishing data trading platforms.

## Mechanism testing

### Transaction cost reduction mechanisms

The development of a digital factor market is beneficial for enterprises as it helps to alleviate information asymmetry and lowers the cost and barriers to accessing information. Additionally, it enhances the value of digital factors, leading to the optimization and transformation of organizational structures within businesses. This paper, uses the management expense ratio as a proxy variable for corporate transaction costs to evaluate whether the establishment of a digital factor market can reduce these transaction costs [17]. As shown in Column (1) of Table 4, the estimated coefficient for the Difference-in-Differences (DID) approach is significantly negative, even when controlling for other influencing factors and fixed effects. This indicates that the construction of a digital factor market does indeed reduce transaction costs for firms. This reduction is likely due to the promotion of uniform data trading rules and standardized contracts, which help decrease the costs associated with information screening and negotiation, thereby lowering overall transaction costs.

**Table 4:** Mechanism test results.

Variables	(1) management cost ratio	(2) R&D intensity	(3) Innovation outputs	(4) Innovation risk	(5) TFP_LP
DID	−0.0093*** (0.0024)	0.0623**(0.0250)	0.0771*** (0.0229)	−0.0013** (0.0005)	0.0389*** (0.0094)
Control	Yes	Yes	Yes	Yes	Yes
Time/firm Fe	Yes	Yes	Yes	Yes	Yes
Observations	20846	16606	20846	15111	20846
R-squared	0.4850	0.7980	0.7393	0.7326	0.9207

### Innovation incentives and risk smoothing mechanisms

The development of marketization in the digital factor market underscores the government’s commitment to advancing the digital economy through a signaling mechanism. The provision of special subsidies and preferential policies effectively lowers the costs associated with enterprise innovation and enhances the efficiency of resource allocation. As a result, companies are increasing their investments in innovation, which significantly boosts their innovation output. In this paper, we use R&D intensity to represent innovation inputs and the natural logarithm of patents granted for inventions as a measure of innovation outputs. The specific regression results can be found in columns (2) and (3) of Table 4. The estimated coefficients from the Difference-in-Differences (DID) analysis are significantly positive and statistically significant at the 1% level. This indicates that the marketization of digital factors encourages firms to allocate more resources to research and development (R&D), thus increasing both innovation inputs and outputs. Ultimately, this contributes to a rise in the technological complexity of firms’ exports.

The construction of digital factor marketization facilitates the establishment of innovation networks through platform effects, promoting collaboration and breakthroughs among companies in high-quality technologies. As innovation risk decreases, firms are more inclined to invest actively in research and development of innovative technologies. This leads to an increase in both the technological complexity and market competitiveness of their exports. This paper references the work of Wang, Y.Z. et al.[29], which indicates that if a firm’s research and development investment exceeds its business revenue in the following year, it is classified as having high innovation risk (assigned a value of 1). Conversely, if the innovation risk is low or non-existent, it is assigned a value of 0, creating an indicator for the innovation risk variable. As demonstrated in column (4) of Table 4, the estimated coefficients of the Difference-in-Differences (DID) analysis are significantly negative, with all results being significant at the 1% level. This suggests that the construction of digital factor marketization enhances the value of data factors, mitigates the risk associated with innovation activities, and allows firms to engage in technological cooperation and allocate innovation resources more efficiently.

### Total factor productivity enhancement mechanisms

The construction of a data factor market increases the technical complexity of enterprises’ exports by enhancing their total factor productivity (TFP_LP). According to the model in equation (6), the total factor productivity of enterprises is calculated. Column (5) of Table 4 indicates that this marketization has a significant positive impact on total factor productivity, with results that are statistically significant at the 1% level. The econometric analysis in equation (6) demonstrates that the improvement in total factor productivity acts as a key mediating variable that drives the growth of firms’ export technological complexity.

## Heterogeneity test

### Enterprise agile responsiveness heterogeneity

Agile responsiveness refers to an organization’s ability to sense and recognize changes in the external environment and respond quickly to meet customer needs [30]. Variations in firms’ agility can lead to significant differences in how data factor marketization policies affect their export technology complexity. Drawing on the methodology of Fan and Pan [31], this study considers board meeting frequency as an objective indicator of organizational responsiveness. As the core decision-making body of a corporation, the board’s convening frequency serves as a visible indicator of strategic engagement. Frequent board meetings typically indicate that the enterprise is actively responding to external changes, conducting concentrated deliberations on new opportunities and challenges, and demonstrating agile response capabilities at the governance level. In this paper, the number of board meetings held during the current period is used as a proxy for corporate agility responsiveness. Firms are categorized into high and low agility responsiveness groups based on whether their levels exceed or fall below the median. The grouping test was conducted on the study sample, and the estimation results are presented in columns (1)-(2) of Table 5. The findings indicate that the coefficients of the core explanatory variables are significantly positive only in the high agility responsiveness group. This suggests that the positive impact of data factor marketization on the export technology complexity of firms with high agility is more pronounced than that of firms with low agility. The likely reason for this is that highly agile firms typically have better governance mechanisms and quicker decision-making capabilities. They are able to recognize opportunities presented by data factor marketization in a timely manner, thereby enhancing the technological complexity of their exports by leveraging the value of data. In contrast, less agile firms face challenges in quickly transforming policy opportunities into productivity due to delays in their decision-making processes. This results in an inability to fully capitalize on policy benefits, leading to a limited impact of data factor marketization on the technological complexity of their exports.

**Table 5:** Heterogeneity test results1.

Variables	(1) High agility response group	(2) Low agility response group	(3) High intelligence level group	(4) Low intelligence level group
DID	0.0064** (0.0025)	0.0043 (0.0029)	0.0026* (0.0015)	0.0079 (0.0071)
Control	Yes	Yes	Yes	Yes
Time/firm Fe	Yes	Yes	Yes	Yes
Observations	10567	9035	16501	3853
R-squared	0.9044	0.9196	0.9370	0.9343

### Heterogeneity in the level of enterprise intelligence technology

According to the resource-based view theory, the advanced technology utilized by enterprises is considered a crucial organizational resource. To unlock the value of data elements through marketization, companies must have the necessary intelligent technology capabilities in place. In this paper, we use the frequency of keywords related to AI technology in the annual reports of enterprises as a proxy for the level of their intelligent technology. We categorize these enterprises into high and low intelligence technology groups based on the median level of technology. If an enterprise’s level exceeds the median, it is classified as a high-level group; otherwise, it falls into the low-level group [32]. The sample was tested for grouping, and the estimation results are presented in columns (3)-(4) of Table 5. The findings indicate that the digital factor marketization policy has a significantly positive impact on the export technological complexity of high-intelligence technology firms. In contrast, the effect on low-intelligence technology firms was not statistically significant. This suggests that the enhancement effect of the digital factor marketization policy is more pronounced in high-intelligence technology firms. One possible explanation for this difference is that high-intelligence technology firms benefit from well-established digital infrastructures and specialized talent pools. These resources enable them to more effectively integrate data elements into their innovation processes, thereby increasing the complexity of their export technologies through a data-driven research and development model. In contrast, low-intelligence technology enterprises often struggle to adapt due to a lack of necessary technology platforms and skilled personnel. As a result, they face challenges in responding to the demand for technological upgrades driven by the marketization of data elements, which limits their ability to leverage the potential benefits of this marketization on the complexity of their export technologies.

### Deep Heterogeneity in Enterprise Intelligence Technology Adoption

The depth of smart technology adoption indicates how effectively firms have integrated technological capabilities into their organizational structures. Variations in this depth of adoption can influence an organization’s ability to transform data elements. To investigate this, this paper uses the number of keywords related to intelligent business found in the annual reports of enterprises as a proxy for measuring the depth of smart technology application. The median value of this depth serves as the threshold to categorize organizations into two groups: those with a high depth of intelligent technology application (above the median) and those with a low depth of intelligent technology application (at or below the median)[32]. The samples were tested for grouping, and the estimation results are presented in columns (1)-(2) of Table 6. The coefficients of the core explanatory variable (DID) are significantly positive for enterprises with a high depth of intelligent technology application. In contrast, these coefficients are not statistically significant for enterprises with a low depth of intelligent technology application. This finding underscores the crucial role that the depth of smart technology application plays in enhancing the value of data factors. Firms with a higher depth of smart technology application can leverage the value of their data more effectively, which subsequently drives the complexity of their export technology. Specifically, enterprises with high intelligence application depth utilize embedded intelligent technology to establish robust data analysis infrastructures and digital organizational structures. This approach effectively reduces the processing costs associated with data elements and enables data-driven technological upgrades, thereby improving the complexity of their export technology. On the other hand, enterprises with low intelligence application depth are unable to fully capitalize on the value of their data elements, as their use of intelligent technologies is limited to auxiliary functions. Consequently, the impact on the marketization of data elements and the complexity of their export technology is relatively restricted.

**Table 6:** Heterogeneity test results2.

Variables	(1) High Intelligence Application Group	(2) Low Intelligence Application Group	(3) High Monopoly Group	(4) Low Monopoly Group
DID	0.0038** (0.0016)	−0.0039 (0.0089)	0.0036** (0.0017)	0.0002 (0.0029)
Control	Yes	Yes	Yes	Yes
Time/firm Fe	Yes	Yes	Yes	Yes
Observations	16534	3730	10351	10118
R-squared	0.9284	0.9115	0.9636	0.8438

### Heterogeneity in degree of industry monopoly

Differences in the degree of industry monopoly, a key characteristic of market structure, significantly influence how efficiently firms utilize data elements and the intensity of demand for those elements. This paper uses the Herfindahl index as a measure of industry monopoly; a higher Herfindahl index indicates a stronger monopoly and less competition within the industry. Industries are classified into high and low monopoly groups based on whether their Herfindahl index is above or below the median. A group regression test was conducted, and the results are presented in columns (3) and (4) of Table 6. The coefficient for the core explanatory variable (DID) is significantly positive in the high-monopoly group but does not pass the significance test in the low-monopoly group. This finding suggests that firms operating in high-monopoly environments can utilize data elements more effectively, primarily due to their superior resource integration capabilities and economies of scale.

The marketization of data factors has lowered the cost of accessing data, enabling high-monopoly firms to efficiently integrate data into their production processes by leveraging existing redundant resources and well-developed digital infrastructures. As a result, this integration increases the technical complexity of their exports. Conversely, while the marketization policy aims to reduce access and transaction costs for data, micro and small firms in highly competitive industries often struggle to benefit. Their size limitations and lack of resources mean that the advantages of using data factors often do not outweigh the associated costs, thereby limiting the positive impact of data factor marketization on enhancing the technological complexity of their exports.

### Research conclusions and policy recommendations

Data factor marketization is a crucial institutional innovation for resource allocation in the digital economy, offering systematic support for data factors in enhancing the technological complexity of enterprise exports. This study addresses gaps in existing research by focusing on three core issues: testing net effects and heterogeneity, identifying mechanism pathways, and overcoming endogeneity. It treats the establishment of data element trading centers as a quasi-natural experiment, employing a staggered double difference approach. Using data from Chinese A-share listed companies from 2012 to 2022, the study examines the impact of data element marketization on the technological complexity of corporate exports. Key findings are as follows:

(1)This paper provide clear empirical evidence confirming the positive net effect of data factor marketization on firms’ export technological complexity;(2)This study systematically identifies four key mechanisms through which data factor marketization affects export technological complexity: reducing transaction costs, incentivizing innovation and mitigating innovation risks, and improving total factor productivity. (3) Heterogeneity test reveals significant differences in how data factor marketization impacts the enhancement of export technological complexity. Enterprises with high agile response capabilities, advanced levels of smart technology, deeper integration of smart technology, and those operating in high-monopoly industries experience more pronounced benefits.

Based on these findings, the paper presents the following policy recommendations:

First, it is essential to improve the institutional framework for the market-oriented allocation of data elements and to establish a national integrated data trading market system. Currently, China’s data factor market faces issues such as fragmented regulations and inconsistent standards, highlighting the urgent need for a national-level institutional structure. At the regulatory level, policymakers should clarify the definitions of data property rights, transaction rules, and regulatory mechanisms. This involves establishing a standardized system that encompasses the entire data rights chain, including valuation and circulation. By providing clear institutional guidance, we can reduce transaction uncertainty and address information asymmetry among market participants. At the level of trading platforms, policies should leverage existing data trading centers to create national-level data circulation hubs. This will enable the integration of interregional data resources and foster a unified operating model characterized by “ unified registration, hierarchical trading, and collaborative supervision. “ Such measures aim to break down data silos and ensure efficient cross-regional allocation of data elements. At the market level, the government should encourage innovative data factor trading models and explore financial tools such as data futures and data asset securitization. By transforming data value into quantifiable and tradable market products, policymakers can stimulate the intrinsic motivation of enterprises to engage in the data factor market.

Second, there is concern about the existence of corporate capacity thresholds and industry barriers that limit the effectiveness of data factor marketization policies. It has been observed that the impact of these policies on enhancing the technological complexity of firms’ exports varies significantly among different types of companies. Firms that exhibit high agility, possess advanced intelligence, and operate in monopolistic industries benefit substantially, while firms with lower capacities struggle to fully capitalize on these policy advantages. This situation raises the possibility of a “Matthew effect,” where the gap in digital capabilities between enterprises could widen. Therefore, it is essential to implement differentiated policies that promote the coordinated development of various types of enterprises. (1) Establish a tiered support system and provide customized policy packages for enterprises with varying capabilities. Specifically, this entails opening government data resource databases and granting pilot qualifications for cross-border data flows to high-capability enterprises. For low-capability enterprises, implement a “data poverty alleviation” plan that includes data literacy training, basic data analysis tools, and subsidies for data applications. (2) Set up a special support fund focused on helping less-capable enterprises improve their data infrastructure and enhance their data application capabilities. This fund can be utilized for purchasing equipment, training talent, and procuring technical services. (3) Create an industry data sharing platform to facilitate the sharing of data resources from high-monopoly enterprises. The government should encourage resource-rich enterprises to appropriately share their data, promoting a reasonable flow of data elements and the realization of value. The implementation of these measures should be accompanied by the establishment of assessment mechanisms and regulatory frameworks to prevent market imbalances. By undertaking systematic measures, the universality of data factor marketization can be enhanced.

Third, it is essential to emphasize the importance of marketizing data as a factor in driving the technological complexity of firms’ exports. Marketizing data enhances this complexity through several mechanisms, including reducing transaction costs, providing innovation incentives, smoothing innovation risks, and improving total factor productivity. Therefore, policymaking should closely align with this fundamental logic. To reduce transaction costs, the government should focus on optimizing the institutional environment and infrastructure of the data factor market. This includes simplifying the approval process for the cross-border flow of data, establishing data trading rules that conform to international standards, and minimizing institutional barriers that hinder enterprises from accessing global data resources. To enhance innovation incentives, relevant departments must improve the intellectual property protection system and strengthen patent protection for data-driven technological innovations. Additionally, the government should establish a special fund to support enterprises in researching the integration of data elements and exporting technology innovations. Furthermore, creating a collaborative innovation platform that connects industry, academia, and research is essential to accelerating the transformation of data elements into tangible productivity. To manage innovation risk, the government should focus on creating a multi-layered system for risk-sharing and collaboration. Establishing a data innovation risk compensation fund and developing data insurance products can help reduce the trial-and-error costs associated with data application for businesses. Additionally, regulatory authorities should build a database of industry data risk cases and implement a monitoring system for cross-border data flows to help companies avoid compliance and technological risks. Encouraging businesses to form innovation alliances through a data-sharing platform can promote open collaboration and diversify research and development risks. To enhance total factor productivity, the departments of industry and information technology need to promote the deep integration of data elements with traditional industries. They should support companies in utilizing intelligent algorithms to optimize production processes and supply chain management. Furthermore, education and human resources departments should work on strengthening data skills training systems to enhance the data literacy of the workforce. Development and reform departments should lead the way in improving data-driven decision support systems to assist businesses in achieving accurate resource allocation. At the same time, local governments can collaborate with industry associations to establish industry-level data-enabling platforms, facilitating the harmonious flow of technology, capital, data, and other resources to fully unlock the potential for productivity growth.

While rooted in China’s specific context, this study’s findings offer valuable implications for global developing nations with diverse digital infrastructure levels seeking to enhance export competitiveness through data factor market cultivation. However, China’s case benefits from a well-established digital infrastructure that facilitates data factor marketization; the core mechanisms identified in this research demonstrate broader applicability across developing economies. For countries with less developed digital infrastructure, priority should be given to establishing institutional foundations for data factor marketization, including the development of data property rights frameworks, the creation of transaction protocols, and the implementation of security systems. We recommend implementing pilot programs in industrial parks or key sectors with relatively advanced digital foundations, adopting a strategy of institutional pioneering to achieve localized breakthroughs and initiate industrial upgrading. As infrastructure continues to develop, data factor marketization will progressively realize its potential to enhance export technological sophistication.

## Supporting information

S1 Data Raw data (.dta). (DTA)

S2 Data Raw data. (.xls). (XLS)

S3 Data Raw Data Manipulation Code (.do). (DO)

S4 Data Results of raw data operations.(.docx) (DOCX)
