# Supplementary material for: Marketization of data elements drives the cultivation of competitive advantages in export products—Based on an export technology complexity perspective
Source: PLoS One. 2026 Feb 9;21(2):e0342262. doi: 10.1371/journal.pone.0342262 (PMC12885324; doi:10.1371/journal.pone.0342262)

Descriptive statistics
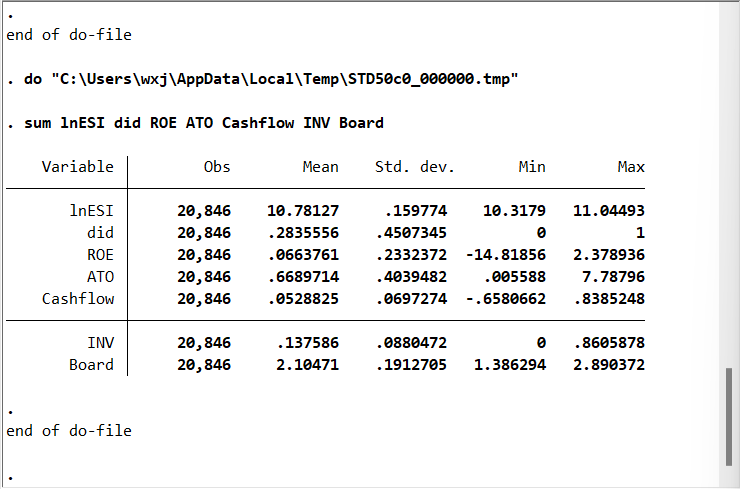


Baseline Regression

Bidirectional fixation
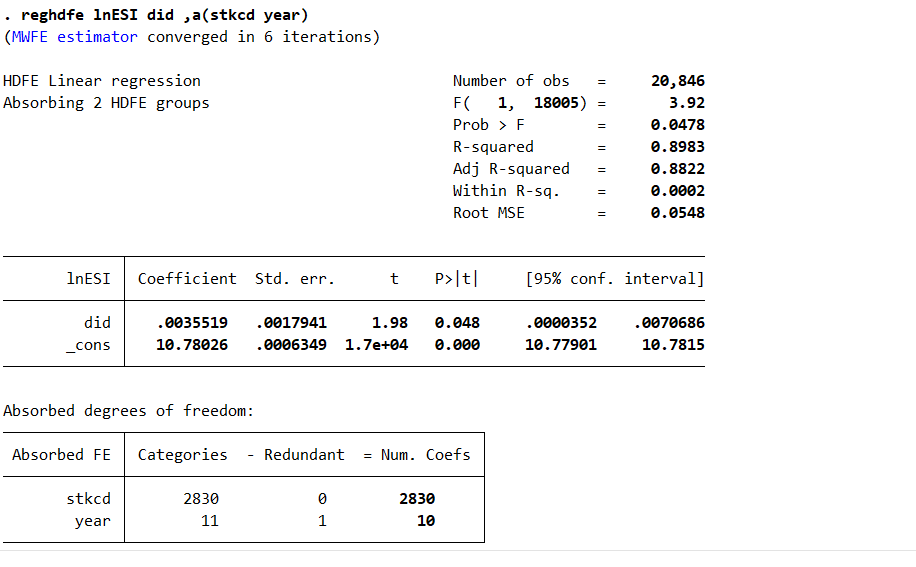
 Time-fixed effect
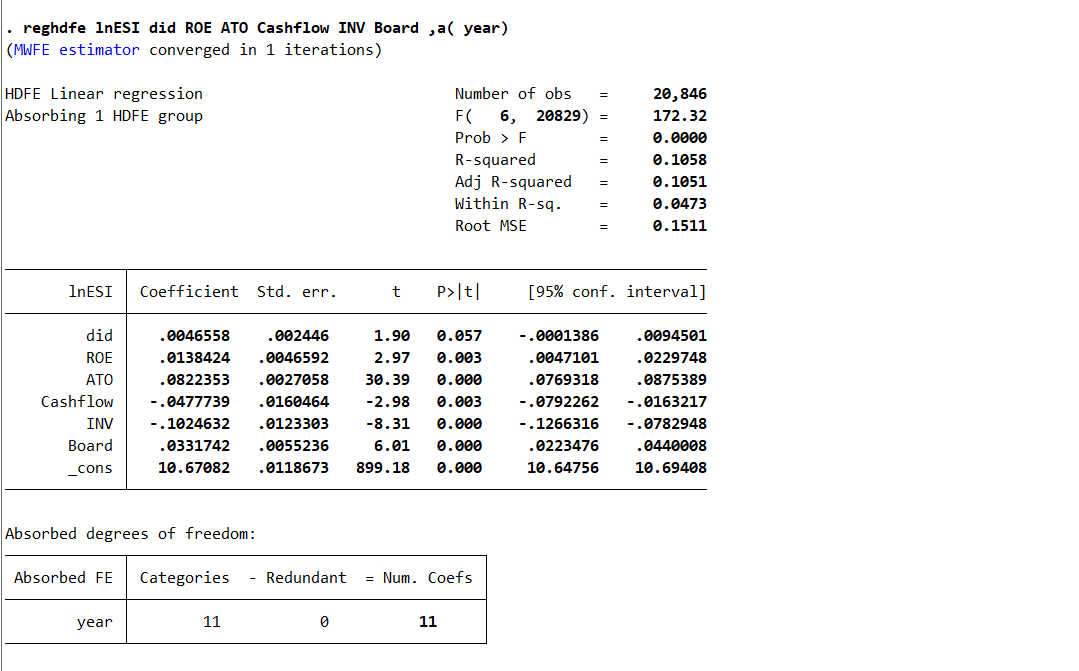
 Individual fixed effects


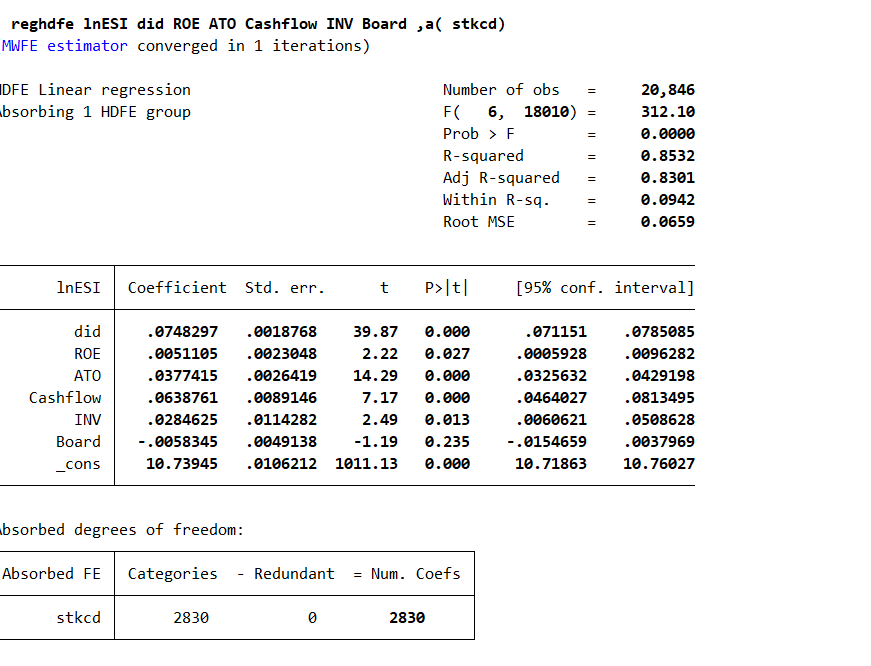
 Bidirectional fixation with controlled variables
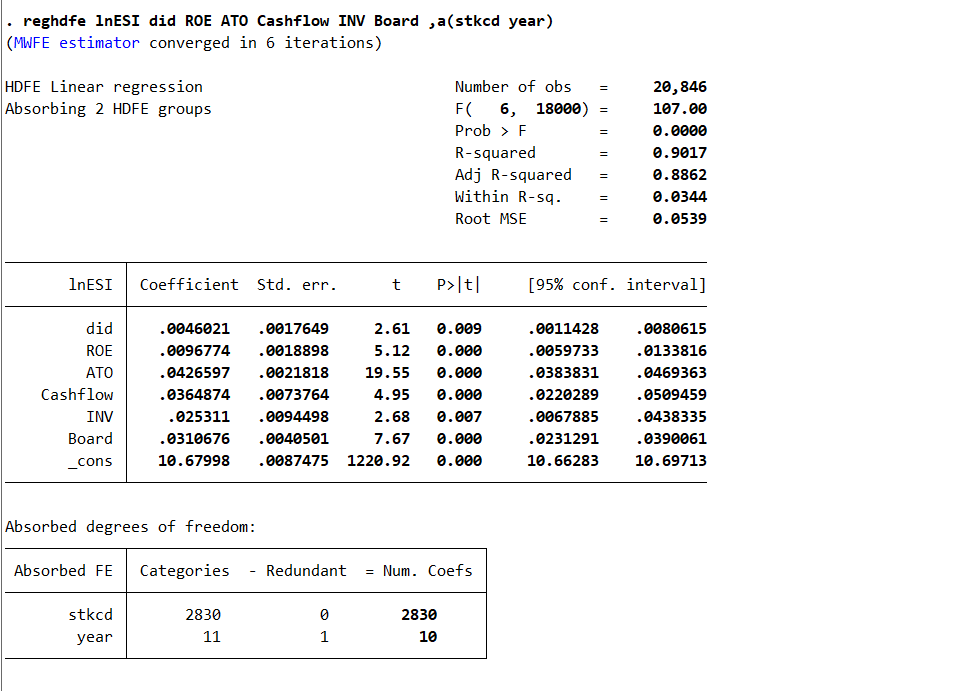


Parallel Trend Test
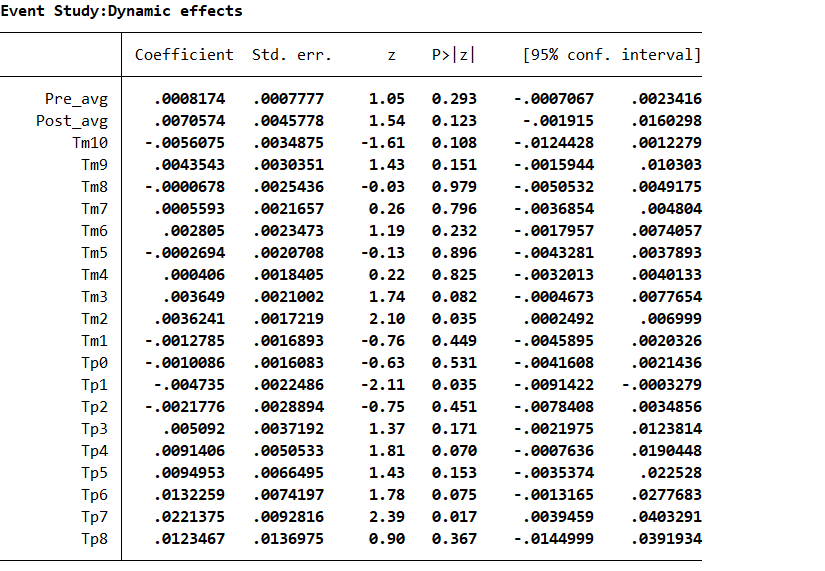


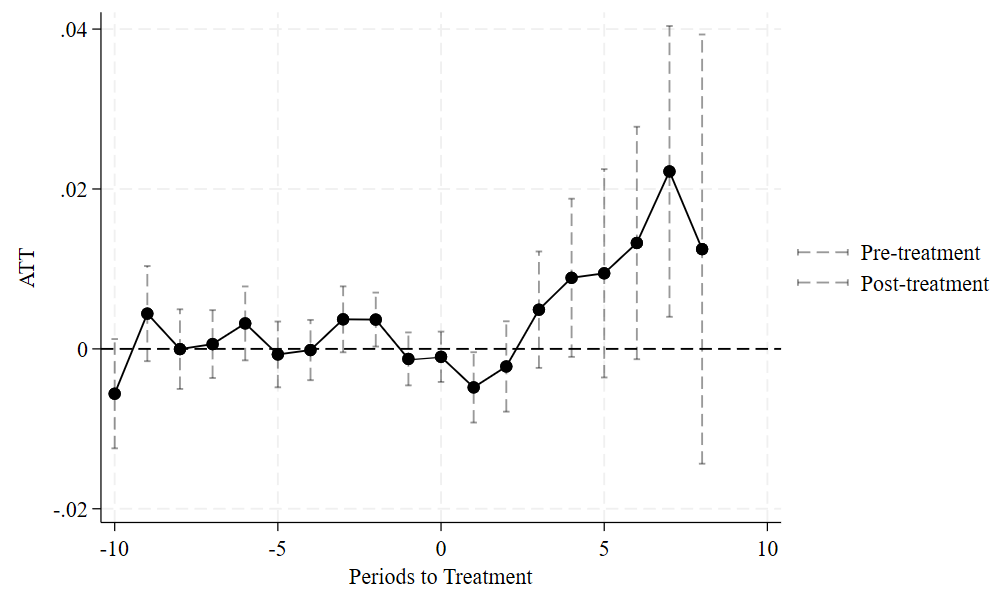


PSM Propensity Score Matching


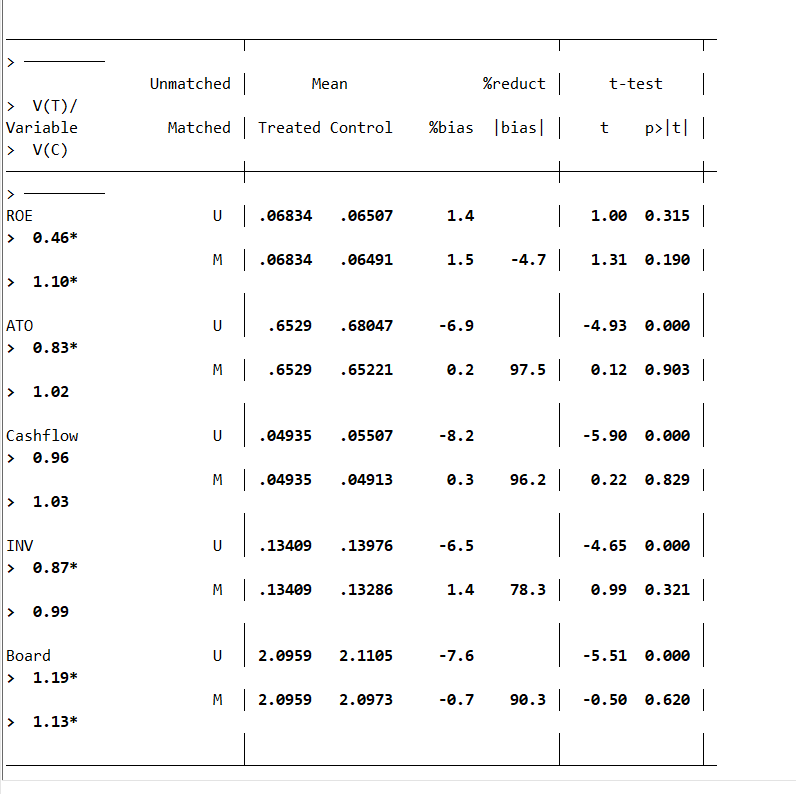


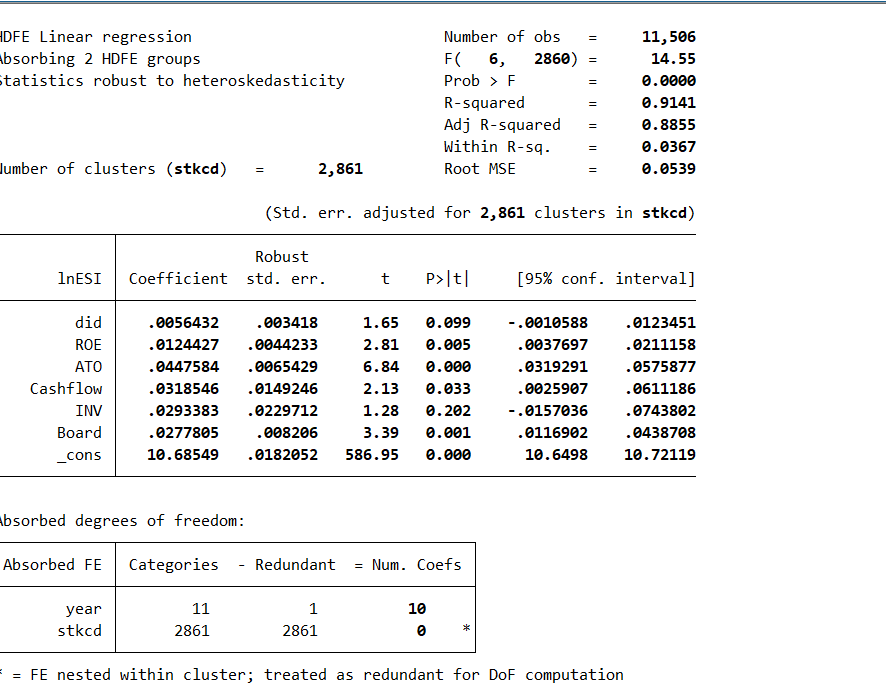


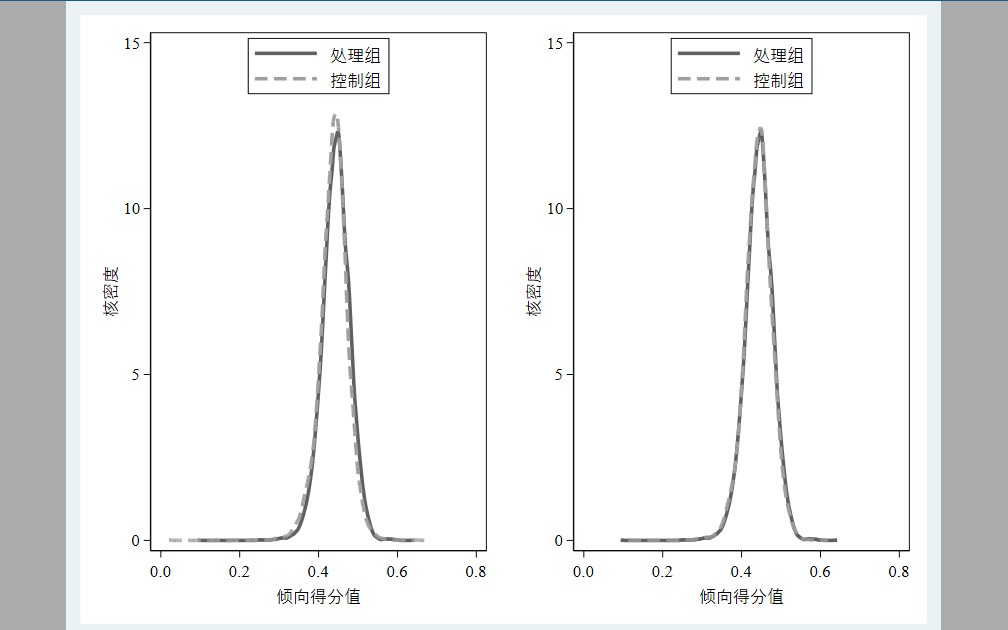


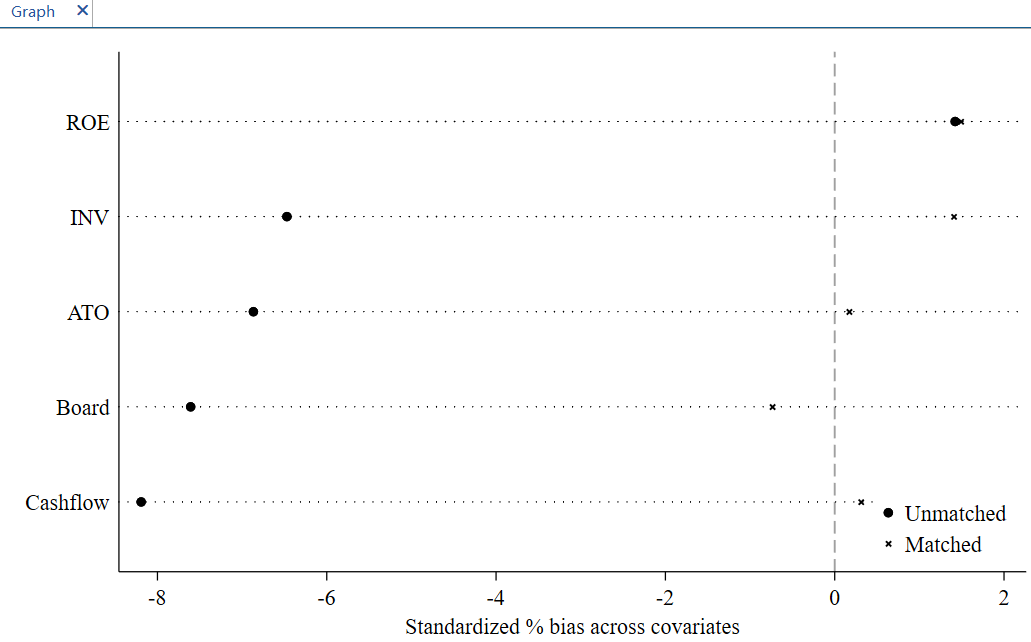


Instrumental Variables Method Tool Variable Method
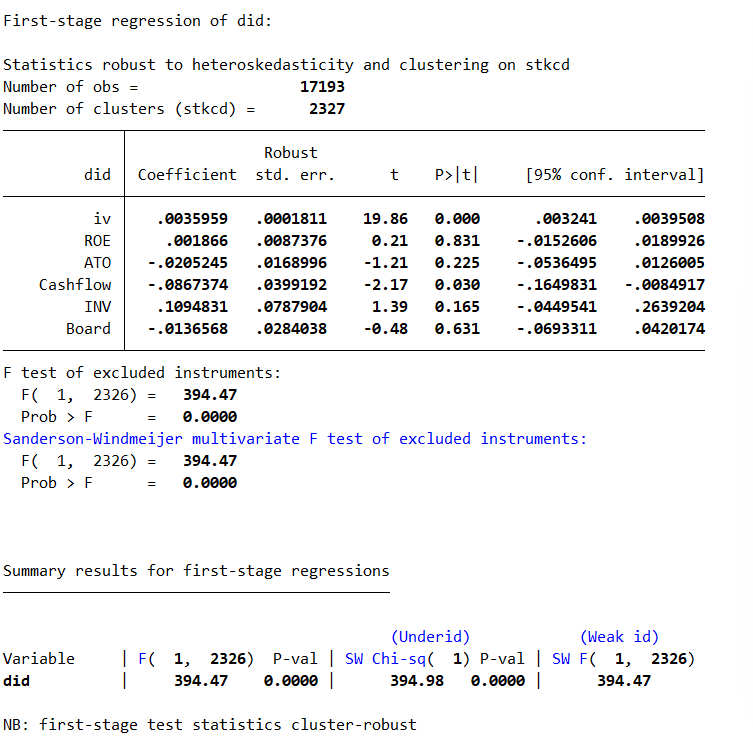


Exclude municipalities
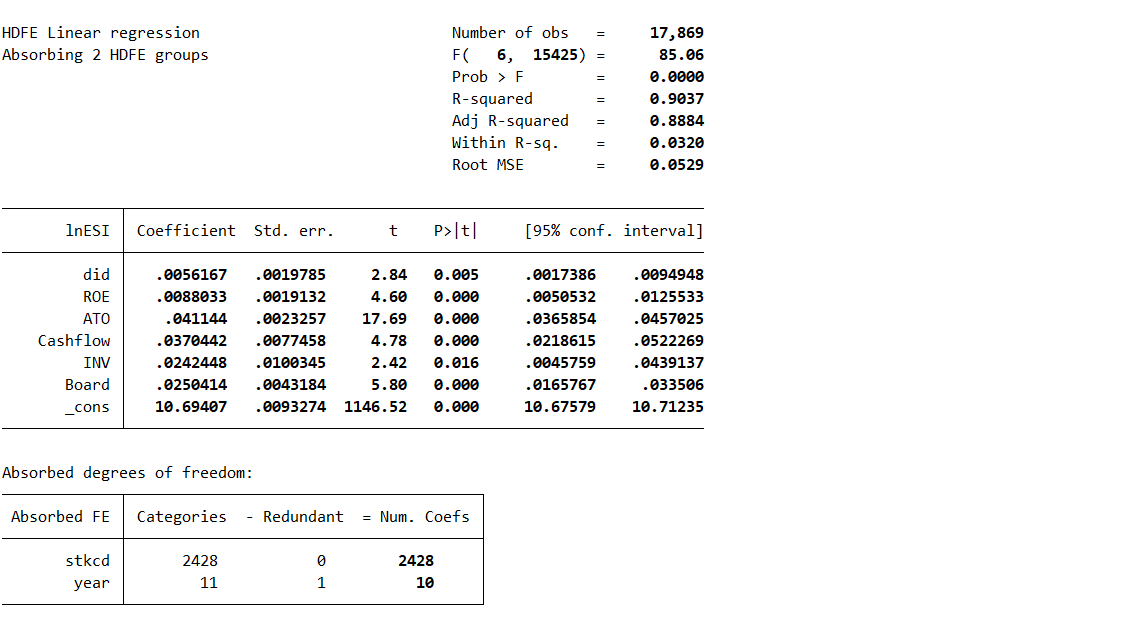


Management Expense Ratio
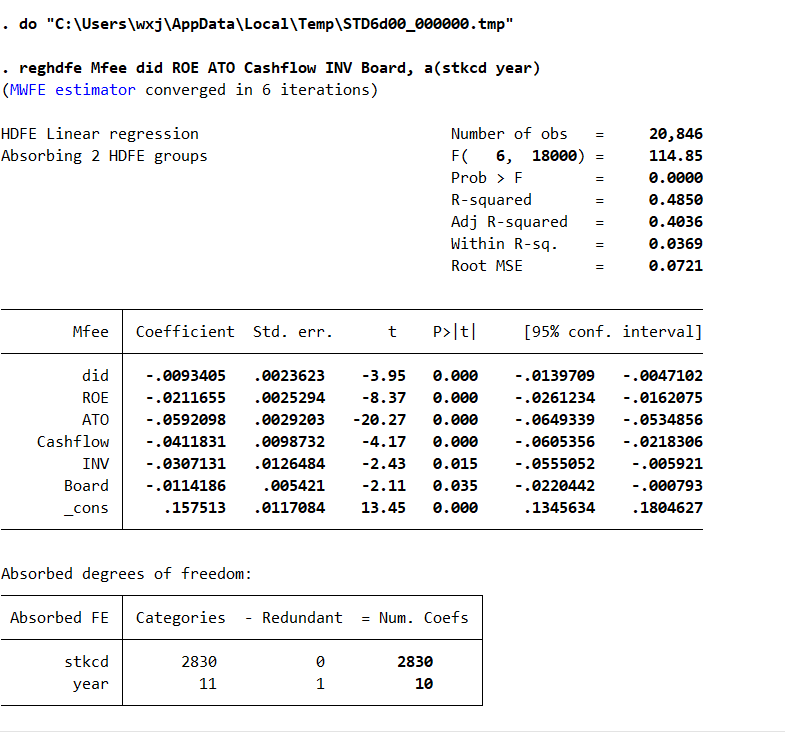


R&D intensity
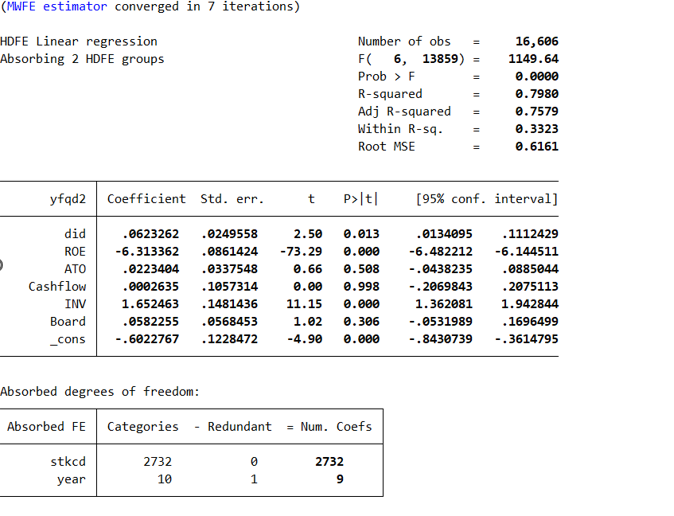


Innovation Output
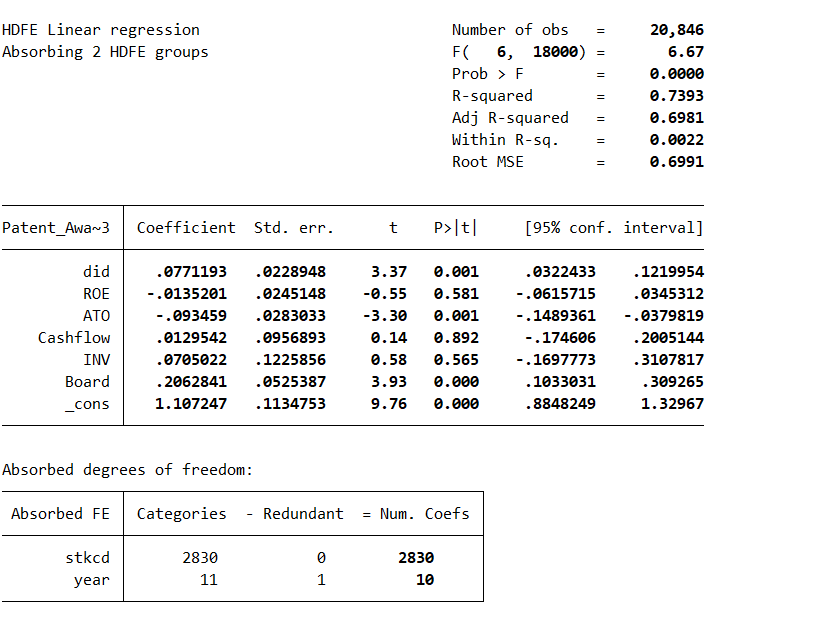


Innovation Risk
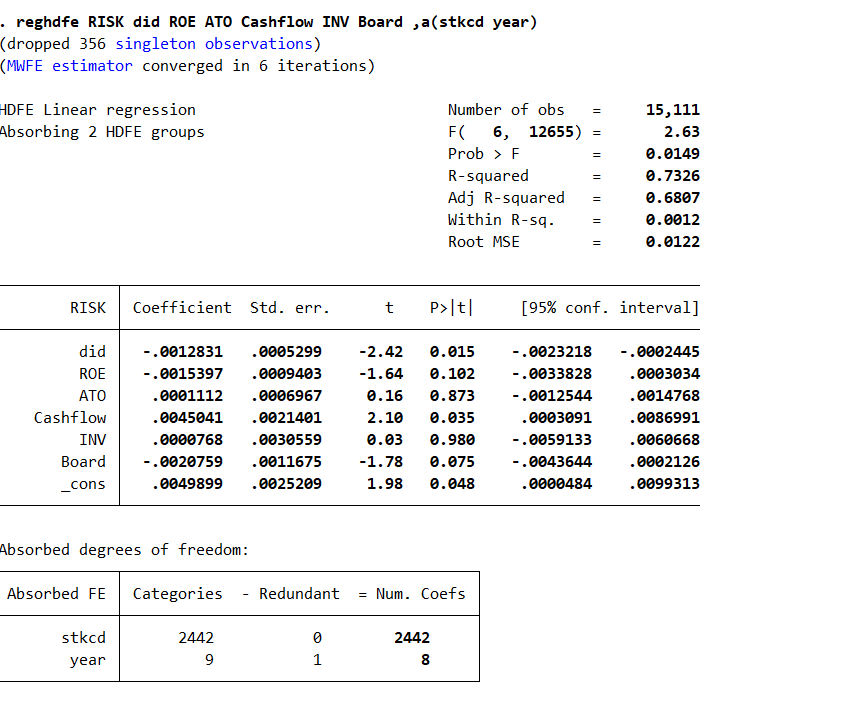


Total Factor Productivity of Enterprises
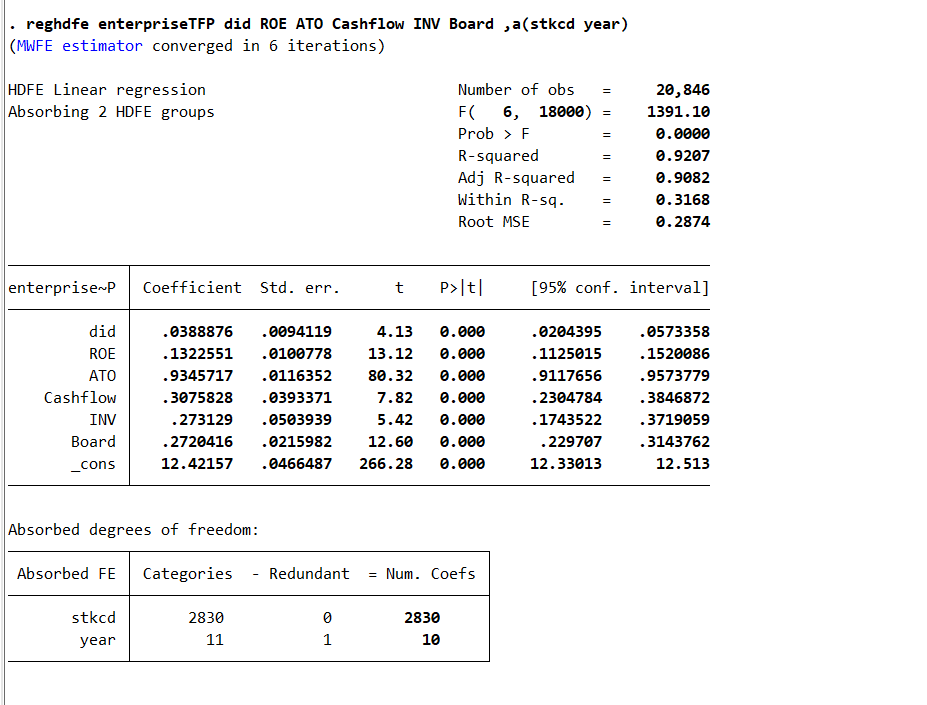


Level of Intelligent Technology
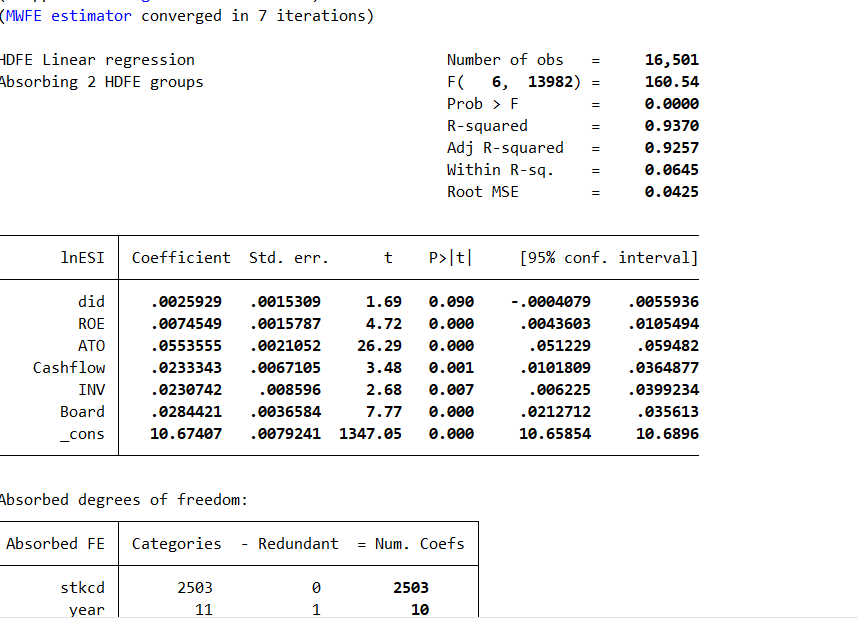

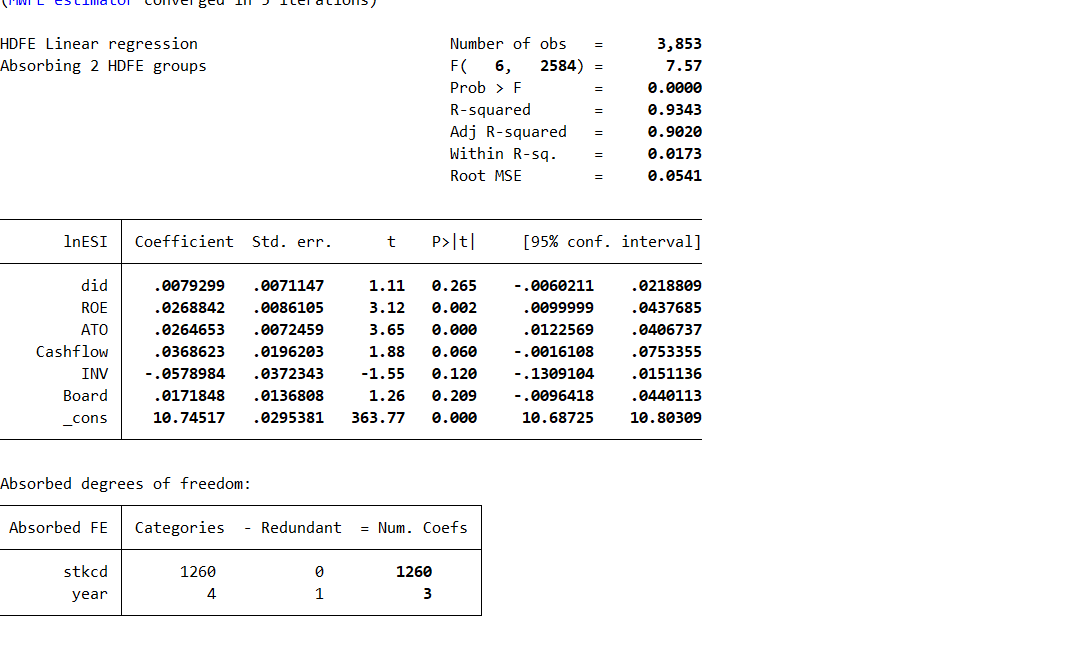
 Level of Intelligent Application
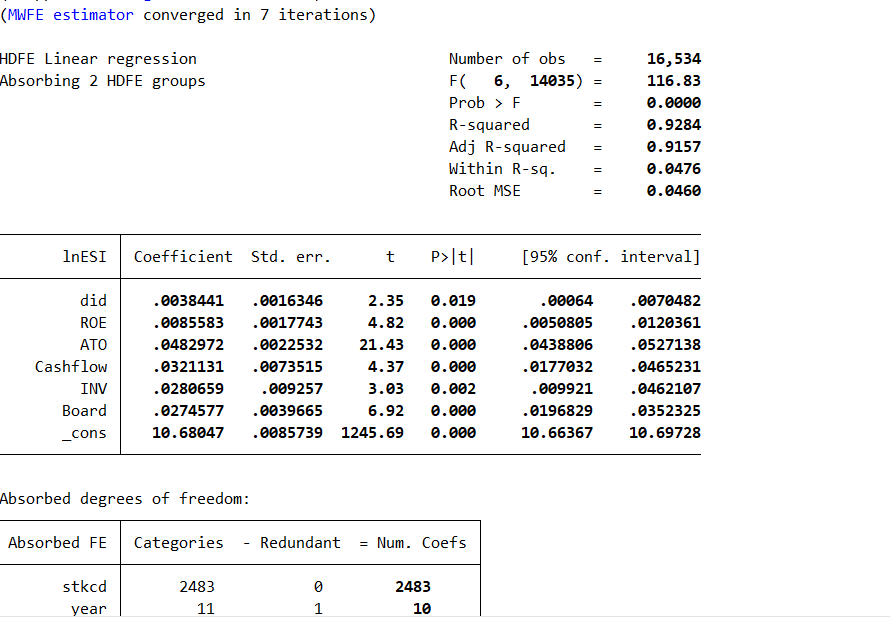

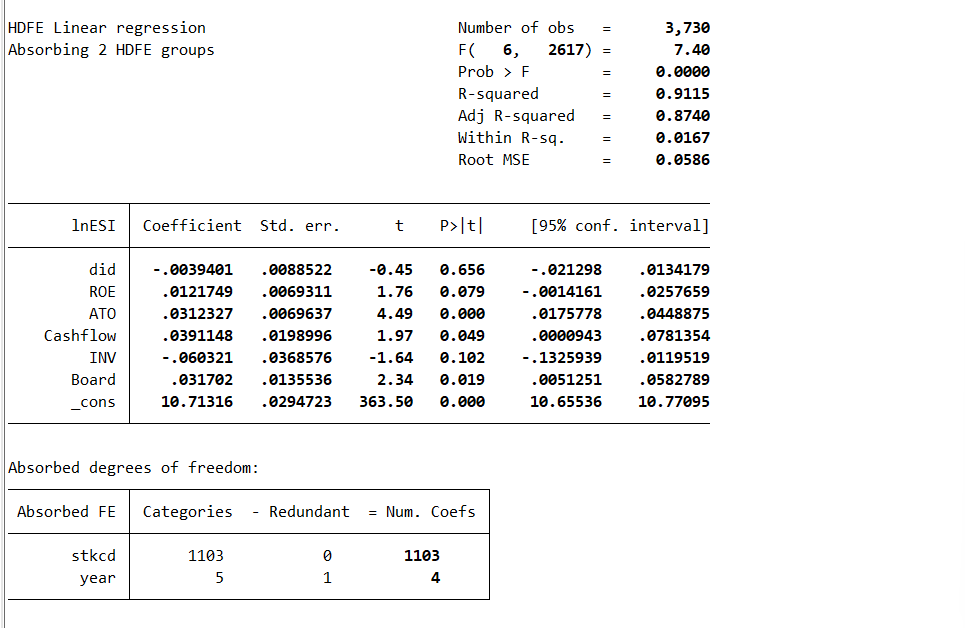
 monopoly


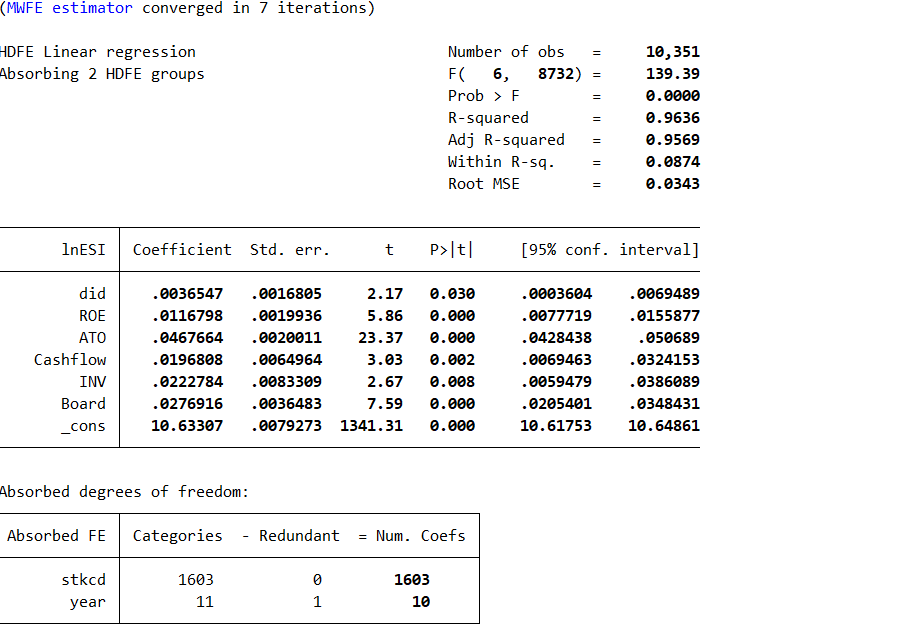

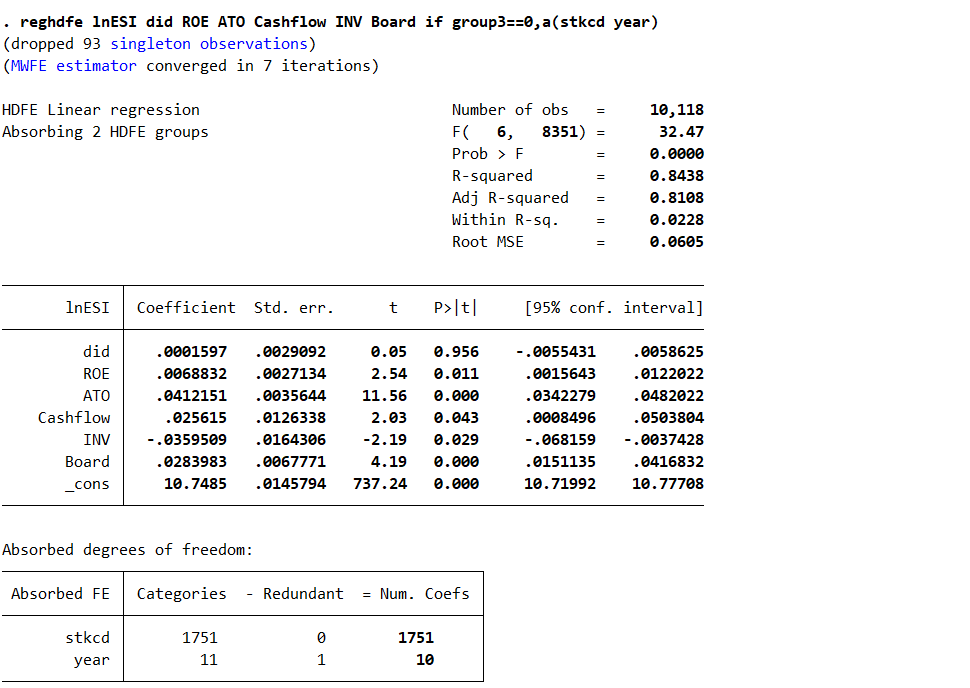


Agile responsiveness
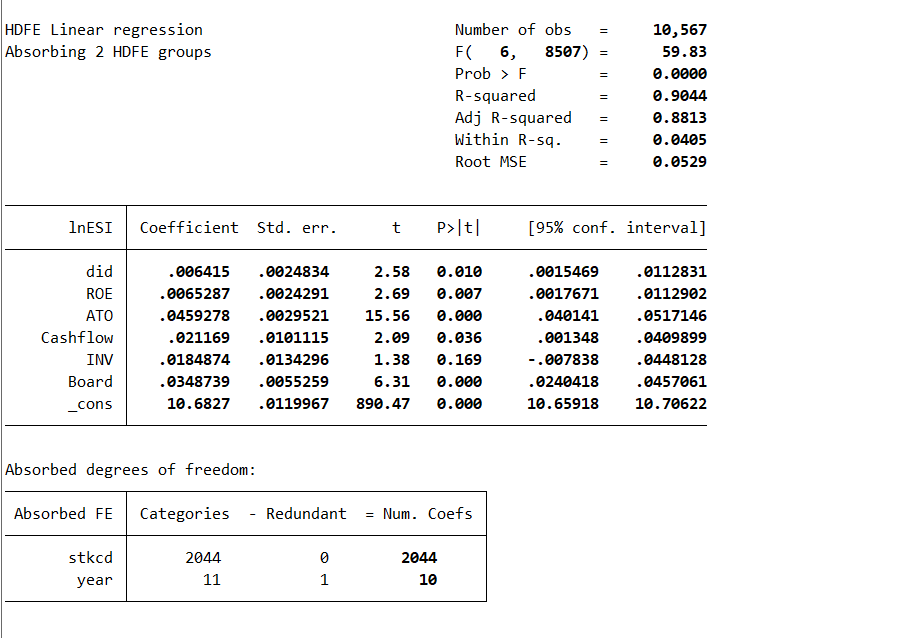

Supplement: S1 Data — (DTA) [file pone.0342262.s001.docx]
